# Challenges for Latina Breast Cancer Patient Survivorship Care in a Rural US-Mexico Border Region

**DOI:** 10.3390/ijerph18137024

**Published:** 2021-06-30

**Authors:** Eunjeong Ko, Veronica Cardenas, María Luisa Zúñiga, Susan I. Woodruff, Viviane Rodriguez, Helen Palomino

**Affiliations:** 1 School of Social Work, San Diego State University, 5500 Campanile Drive, San Diego, CA 92182-4119, USA; mlzuniga@sdsu.edu (M.L.Z.); swoodruff@sdsu.edu (S.I.W.); 2 Department of Psychiatry, University of California, San Diego, Moores Cancer Center, 3855 Health Sciences La Jolla, San Diego, CA 92093-0658, USA; vcardenas@health.ucsd.edu; 3 MSW, Imperial County Behavioral Health Services, El Centro, CA 92243, USA; viviane3407@gmail.com; 4 LCSW, Cancer Resource Center of the Desert, El Centro, CA 92243, USA; hpalomino@crcdinc.org

**Keywords:** survivorship care plan, Latina, rural, breast cancer, US-Mexico border

## Abstract

Rural US Latina breast cancer patients experience language barriers, health literacy issues, and limited access to health care resources that negatively impact survivorship care. This study explored the challenges to survivorship care for rural Latina breast cancer (BC) patients and approaches to supporting survivorship care plans (SCP) from the stakeholders’ perspectives. Data were collected via eight focus groups (n = 40) and individual interviews (n = 4) with Latina BC patients, family caregivers, and health care professionals in a rural US-Mexico Border region. Interviews were audio-taped, transcribed, translated, and analyzed using thematic analysis. Themes related to the patient’s SCP challenges included: (1) lack of knowledge of treatment information, (2) lack of proactive health behavior, (3) gaps in information for care coordination, (4) psychological distress, and (5) difficulty retaining health information. Respondents expressed that the SCP document could fill patient information gaps as well as support patient communication with their clinicians and family. Rural BC patients demonstrated an acute need for information and active engagement in their survivorship care. The findings indicate the importance of addressing challenges for survivorship care on multiple dimensions: Cognitive, behavioral, social, and structural. Developing a culturally tailored SCP intervention will be imperative to support survivorship care.

## 1. Introduction

Breast cancer (BC) is the second most common cancer diagnosis followed by skin cancer among women in the United States [1]. There are currently over 3.8 million breast cancer survivors in the US [2]. Follow-up care for the detection of cancer recurrence, management of long-term effects, preventive screenings for additional malignancies, and other preventive care are extremely important to promote improved health outcomes and wellbeing of cancer survivors [3]. Racial/ethnic disparities in breast cancer are persistent. The incidence of BC and mortality is lower among Latinas as compared to non-Hispanic White women and they have similar 5-year survival rates at 88.9 and 89.6 per 100,000, respectively [4]. From 2009–2018, the BC mortality rate decreased by 1.1% per year among Latinas as compared to a 1.3% decrease among non-Hispanic White women. [5]. Latinas are less likely to be detected for BC at early stages as compared to non-Hispanic White women. From 2009 to 2018, 58.9% of Latinas as compared to 66.5% of non-Hispanic White women were diagnosed at a local stage of BC [5].

Breast cancer is the leading cause of cancer death for Latinas [6]. Latina BC patients, as compared to non-Latinas, have less survivorship related knowledge, are less satisfied with BC care information, and have lower perceived self-efficacy in patient-physician interactions [7]. Latinas with BC also report unmet physical symptom management, poor mental health (i.e., depression, anxiety, stress) [7,8,9], and lower quality of life than non-Latinas [8].

In order to promote successful transitions to survivorship, the Institute of Medicine (IOM) recommends that oncology clinics provide survivorship care plans (SCP) to patients when they are in transition from specialty to primary care. The SCP is a personalized document completed by health care providers that includes a comprehensive summary of clinical and treatment history, care coordination, and follow-up care plans [10]. Previous studies report that BC patients who received SCPs had greater knowledge of follow-up care and greater efficacy in patient-provider communication [11] and adherence to medical recommendations [12].

While the need for SCP remains high, there is a lack of SCP resources in rural communities. Barriers to survivorship care among Latina cancer patients in rural, underserved areas include lack of transportation, financial and insurance issues, language barriers, low health literacy, and limited social and emotional support [13,14,15]. Disproportional cancer care resources, geographic distance, and structural barriers delay or impede their seeking timely cancer care [16,17]. Earlier studies have found that Spanish speaking BC Latina patients were less likely to receive cancer follow-up care, were more depressed, and in need of clinical information (i.e., side effects) as compared to English speaking BC patients [9,18], yet they did not report their psychological issues [19]. While these studies document problems in cancer care among Latinas, little is known about survivorship care issues, particularly regarding concerns and needs for survivorship care among rural Latina BC patients living in the US-Mexico border region. This region is geographically unique as the cancer patients predominantly speak Spanish, have low socioeconomic status, and may access health care systems in both the US and Mexico to complement health care needs and make care more affordable [14,17,20].

The purpose of this study was to obtain diverse perspectives (Latina BC patients, family caregivers, and health care providers) on challenges to survivorship care among Latina BC patients, and the perceived usefulness of the SCP to support their survivorship care planning in a rural, US-Mexico border context.

## 2. Materials and Methodology

### 2.1. Study Design and Setting

The current study was a part of a larger study to develop and test a survivorship care intervention for rural Latina breast cancer survivors (*Proyecto Mariposa*). The current study utilized qualitative methods, focus groups, and individual interviews with clinical and community stakeholders (n = 44) in a rural Southern California-Baja California, Mexico border region. This study region is an agricultural area where the majority of the population is Latino (84%) and more than half of Latinos (52%) have a household income of less than USD 50,000 [21]. The study region is medically underserved with only two medical oncology clinics and one radiation clinic in the 4500 square mile county.

### 2.2. Participants and Recruitment

To elicit diverse perspectives, we purposely sampled different groups of stakeholders involved in the care of cancer patients in this rural region. The group of stakeholders included Latina BC patients who had completed definitive treatment (i.e., surgery, chemotherapy, and/or radiation treatment) and were transitioning to primary care (n = 12), family caregivers (n = 8), and health care providers (i.e., social workers, nurses, medical assistants, and physicians) (n = 24). Cancer patients, family caregivers, and social workers were recruited from a partnering community-based cancer organization that serves a predominantly Latinx population. Health care providers including nurses, medical assistants, and physicians were recruited from an oncology clinic and primary care clinics in this border community. In total, 44 stakeholders participated in the study.

### 2.3. Data Collection

Data were collected from November 2018 to May 2019 via focus groups and individual interviews by the researchers using semi-structured interview guides. We conducted a total of eight focus groups as follows: Two groups with Latina BC patients; two with caregivers/family members of BC patients; two with nurses, and one with medical assistants; and one with social work patient navigators. Interviews with physicians (two oncologists and two primary care physicians) were conducted either in person or by phone. Sample questions included: (1) What have you recognized as BC patients’ needs for survivorship care? (2) What do you think the benefits are of having an SCP? (3) In what way would an SCP be helpful? The interview guide questions were similar in scope but tailored to the respondent type. For example, when asking about the patients’ psychosocial and proactive behavioral issues for survivorship care, the patient focus group questions were “What might you use this for?” and “How might an SCP help you address your concerns regarding communication with the providers?” For provider interviews, we tailored the question for the physicians as health outcome specific: “What health or behavioral outcomes, if any, would you expect for patients as a result of disseminating an SCP program?”

Focus groups with patients and family caregivers were conducted in Spanish, and focus groups and individual interviews with health care professionals were conducted in English by the researchers (EK, VC, and MLZ). Each focus group lasted about 1–1.5 h and individual interviews with physicians lasted about 20–25 min. All focus groups and individual interviews were audio-taped, transcribed, and translated by study research assistants for data analysis.

### 2.4. Data Analysis

We applied thematic analysis [22] to analyze the focus group data. The analysis focused on identifying the nature and impact of different challenges to survivorship care among Latina BC survivors and on the potential usefulness and role of the SCP document that could address these challenges. All the research team members (EK, VC, MLZ, and SW) reviewed the focus group transcripts for accuracy, and read the transcripts while making notes on potential codes. With a discussion of codes, we reached a consensus and developed an initial coding scheme. As each study team member reviewed the transcripts and applied the coding scheme, we used an iterative process to refine the codes and label emerging themes. Each coder then summarized their interpretation of the themes and the research team met to discuss them and select representative quotes for each theme.

### 2.5. Ethical Considerations

This study was approved by the Institutional Review Board (IRB) of the San Diego State University (approval number: HS-2020-0269). Participants were informed of the purpose and procedures of the study. They were also informed of the voluntary nature of the study and their right to withdraw or stop participating in the study at any time.

## 3. Results

### 3.1. Characteristics of Participants

Participant characteristics are presented in Table 1. The average age of participants across the stakeholders was about 46 years (SD = 14.5). For patients, the average of time since completing the definitive cancer treatment (i.e., surgery, chemotherapy, radiation) was 15.6 months. Patients and caregivers all self-identified as Latino/a, and the majority (70.8%) of clinicians reported to be Latino/a, as well. The average of the health care professionals’ (HCP) experience working in cancer care was about 10.6 years.

### 3.2. Qualitative Themes

Common themes across the stakeholders are summarized below. Each theme addressed barriers/challenges related to the patient’s survivorship care and the usefulness of SCP to address these gaps. Primary themes were: (1) lack of knowledge and information needs, (2) lack of proactive health behavior, (3) gaps in information in care coordination, (4) psychological distress, and (5) difficulty of retaining information. Additional quotes for each theme are reported in Table 2.

#### 3.2.1. Lack of Knowledge of Treatment Information

Participants expressed concerns about BC patients’ lack of knowledge or insufficient understanding of their cancer diagnosis, terminology, and the functions of medication. Patients relayed their difficulty in understating medical information. “*I think it (SCP) would be very good because sometimes when you go to a new doctor or something and ask them for the information sometimes the names [terminology] are strange and sometimes they are hard to learn …* ” (P 29). A family caregiver also described the perceived usefulness of SCP for containing essential information.

“When she has another appointment or another surgery to be done for something whatever other surgery is going to happen. You take it with you and show it this … it’s all right there written, the medication the type of cancer what they did, or which doctor did it. Everything will be there”.

(FC 34)

HCPs also voiced concerns about the patients’ medication management. Some patients do not know the difference between the generic and brand name of medication, resulting in taking a double dose. “*They come in with a big bag of meds. Sometimes they have the generic of (medication name) and they’re taking two of the same medication*” (HCP 6). HCPs considered that the provision of knowledge on one’s cancer care “empowers” the patients for the management of illness and perceived SCP as beneficial in enhancing the patients’ understanding of their cancer treatment. “*Knowledge is power. They [patients] have little control over anything so the ability to streamline everything that they’ve gone through is so powerful”* (HCP 1).

#### 3.2.2. Lack of Proactive Health Behavior

The patient’s lack of proactive health behavior such as asking questions of the HCP and managing their illness was found to negatively impact the patients’ survivorship care. It was observed that despite the symptom burdens and ongoing treatment-related issues, patients frequently withhold sharing their concerns with their HCP. “*One [patient] needs to ask [questions] and sometimes you do not know what to ask or how to ask …*” (P 31).

Caregivers emphasized the SCP as providing the necessary information (i.e., side effects), and helping the patients and family to be proactive in asking questions. “*Some people had their hair but not my mom. Her hair fell out and her teeth broke. This [SCP] helps us. I was going to discuss this because doctors often do not [discuss]”* (FC 17). An HCP elaborated, “*They can see and check off what’s relevant to them and what they’d like to have it filled in if there is something missing in their treatment plan*” (HCP 44). Living adjacent to the Mexico border, some patients frequently travel or move to Mexico after the treatment ends which complicates the US clinic’s contact with patients and care continuity. Under these circumstances, the SCP can provide critical information to clinicians who had not been involved with the patient’s care.

*“We live so close to the border that sometimes you know people [patients] just move to Mexico and we don’t know what happened to them. If they know that there are actually more that they need to do [treatment], then perhaps they’ll stick around”.*

(HCP 25)

#### 3.2.3. Gaps in Information for Care Coordination

Participants addressed the lack of patient information shared among HCPs, negatively impacting the patients’ transition to survivorship care. A patient shared her experience on the gaps of information transfer among the clinics. “S*ometimes my doctor had no information of the treatments provided by another doctor. Even the specialist told me that they have not sent it …”* (P 29). The SCP was seen as filling the information gaps among HCPs, and to be more valuable when patients encounter medical crises or adversarial situations (i.e., “accident”) when their information should be readily available to the HCP.

For family caregivers closely involved in patient care, the SCP could become a useful source for the family to present patient information.

*“In my case, my sister who had cancer takes care of my mom. She brings her [mom] to the doctor. When I took her [mom] to her appointment* *because my sister had a surgery on her cancerous tumor* *, I did not know whom she’s been seen with. They asked us for her doctor’s name. I didn’t know because my sister always took her … it [SCP] would be very helpful to look at, like what doctor they’re going to have an appointment with”.*

(FC 17)

In receiving other specialty care or transition to primary care, the lack of patient information can delay treatment which is frustrating for both the physician and patient. This is more imperative for the patients who seek health care outside of the border town. With the lack of a centralized electronic system, the SCP can fill the information gaps, resulting in improving the patients’ continuum of care.

*“They [patients] travel to San Diego, Arizona … then they get sick. Having the folder would be very helpful you know, like their profile, because I know we don’t have electronic [record] for everybody to access”.*

(HCP 43)

#### 3.2.4. Psychological Distress

Participants recognized the patients’ emotional and psychological distress related to the cancer diagnosis and treatment that could impact their survivorship care. The inability to disclose their cancer diagnosis due to the fear of upsetting family members was found to increase pressure and burdens on the overall management of cancer care.

*“I did not tell my mom. I secretly had the operation and I went to recovery hidden from my mother. I live with her. She is a very old lady. If I were to tell her [about this], she could be devastated. So, everything was covered with layers for a month, that’s how long it took for the recovery. After the chemo I would lose my hair then I invent one thing … I spoke to the oncologist and she told me, ‘Go to the psychiatrist tomorrow because it is not possible to do what you’re doing’”.*

(P 30)

Some patients were reluctant to follow up with the doctor for fear of having negative results and causing stress. A caregiver noted:

“*They don’t wanna’ go to doctors and get checked up because they don’t wanna’ know anything … if they find out if they have it [cancer], they are going to think about it, stress out and worry and they’re going to wind up getting more sick than just by the cancer*”.

(FC 34)

By providing information on the cancer treatment history and future plans, the patients viewed SCP as easing psychological distress and providing “peace of mind”. The HCP’s perceptions about the usefulness of SCP focused more on its role in demystifying the information which would reduce their anxiety.

“*When patients hear cancer, a lot of them think this is terminal. They don’t know what to do … they don’t exactly know what’s going on with them and what is going to happen. I think just by knowing more about their disease, their condition, and what they’re taking, their mental state can be better. They can have a little more idea of what’s going on with them*”.

(HCP 9)

#### 3.2.5. Difficulty Retaining Health Information

Participants addressed the patients’ difficulty of retaining information and forgetfulness as a challenge for their survivorship care. Memory lapse might be related to cancer treatments, as one patient shared: “*I don’t know if it’s because of the treatment, but I forget everything*” (P 11).

Family caregivers perceived that patient information being readily accessible in the SCP can help them better manage their patient’s care. A caregiver emphasized how SCP might help her know her daughter’s information without feeling bad about asking multiple questions to her daughter.

“*I would accompany my daughter [patient] to her appointment and I end up not remembering the names of the doctors and others. To be honest, I have a horrible memory. This is very good because sometimes I ask her what doctor are you going to see … she would tell me but many times I would avoid talking with her because I felt bad …*”.

(FC 16)

HCPs believed that the SCP would aid in filling memory gaps, providing all the necessary information, and functioning as a reminder for the patients’ follow up care (i.e., medication management).

“*In the future, I mean if something happens, this will be the way they can tell their doctor ‘you know this was given to me, this happened to me’. Because it might happen that so many years up you won’t remember anything, so that might be their information*”.

(HCP 5)

## 4. Discussion

Our study findings identified important knowledge gaps in cancer care treatment, information needs for Latinx survivorship care, and psychological distress issues that could impede effective survivorship. Similar to the findings from other studies [23,24], patients lacked cancer information. The participants, particularly HCPs, were concerned about their rural Latina patients’ ability to understand their diagnosis and medication management. As addressed in our study, obtaining and processing medical information might be hindered by a language barrier and health literacy barriers. Low health literacy among rural residents is a significant issue such that cancer patients in rural regions had a 33% likelihood of lower health literacy as compared to their urban counterparts [25]. The SCP was perceived to be important to fill the informational needs which is critical for successful recovery after treatment, which has been found in previous studies [26]. This finding amplifies the need for the development of a linguistically and culturally relevant SCP for Latina BC patients.

Our participants perceived that the patients’ lack of proactive behaviors, such as information seeking behaviors, and monitoring and adherence to follow-up care, posed barriers to optimal survivorship care. This result is consistent with previous studies [27,28] which noted the lack of proactive behaviors among Hispanic cancer patients. Our patient participants recognized the importance of asking questions yet expressed difficulty on what and how to ask questions. Torres et al. [29] also found that Latinos, as compared to non-Latino Whites, are significantly less likely to know how to ask questions about their own health. Our participants offered various explanations. For example, lack of cancer-related information and psychological distress were seen as impediments to gathering, processing, and organizing their thoughts in order to ask questions. Previous studies identified patient-physician language concordance, patient’s health literacy, education and acculturation impacting the patient-provider communication and information seeking behavior [30,31]. In a rural region with limited health care resources, the practitioners’ time constraints are a challenge as they struggle to manage the high volume of patients. Having access to the necessary information in the SCP, our participants believed that the SCP would support patients in using the information to ask questions of their clinicians and be able to better manage their own cancer care. The SCP can function as enhancing confidence and activating the patients’ behaviors for survivorship care [32].

The lack of communication between providers regarding their patient’s medical information was perceived to negatively impact patients’ survivorship care, particularly during the transition to primary care. Participants agreed that the SCP can bridge information gaps between providers and prevent the delay of patients’ treatments. The improved transmission of patient information may be even more imperative for rural cancer patients who often travel outside of the community to a neighboring US city or crossing to Mexico for their medical care due to the limited health care resources in this rural region [16,17]. For those who receive health care from multiple providers including those out of the state or county, the SCP document has the potential to convey critical health and treatment information to providers. In a previous study [33], primary care physicians identified SCPs as helpful in saving time for providers in order to obtain the patients’ medical information such as cancer treatment history and recommendation for follow-up care.

Our findings underscore the need for psychological support for Latinas as they navigate options regarding cancer care and consider the potential for cancer recurrence. Fear of cancer recurrence is one of the greatest concerns among cancer survivors [34,35] and can be intensified by less frequent visits with their oncologist post treatment. Fear and avoidance of receiving negative results could also impede follow-up care among some patients resulting in increased stress and potentially causing more illness. The usefulness of the SCP was emphasized in relation to its role in accessing information and clarifying cancer information. The patients’ clear understanding of what follow-up care entails and its timing could lead to reducing the patients’ misinformation and confusion, which contributes to psychological distress and fear of cancer recurrence.

Patients in this study expressed difficulty in retaining or recalling their cancer-related information, and memory problems related to the cancer treatment (i.e., “chemo brain”) are not uncommon among cancer patients [36]. There was consensus among our participants that the SCP functions as a memory aid, filling in gaps of information that can assist patients in adhering to follow-up care. The SCP was also seen as potentially helpful for families to better care for their loved ones by providing important information related to treatment side effects and symptoms. Having family support among Latina BC patients is highly valued and was found to be a positive coping strategy [37]. In this rural setting, the family’s involvement with patient care is critical as they provide an array of support including aiding in communication with the patient’s HCP. Hence, the SCP could further facilitate family caregiver communication with primary care clinicians regarding their loved one’s cancer treatment history. Data collection for this study took place prior to COVID-19, and given the disproportionate impacts of COVID-19 on Latino health and care seeking behaviors, future studies may include assessing lingering impacts of COVID-19 on survivorship care.

Our study has several limitations to note. Our study sample is limited to a Southern California/US Mexico border region, and as such, participants may not represent Latinos in other border regions or in the US. A larger sample of patients and providers across the US border with Mexico may allow for a greater understanding of Latina BC patient survivorship care planning needs. These qualitative research findings do, however, provide insights into concerns faced by Latina BC patients and gaps in survivorship care planning to inform avenues for developing culturally tailored SCP interventions for Latina BC patients.

## 5. Conclusions

In response to the growing concerns over health disparities in cancer survivorship care among Latinas with BC, this study sought to understand the perspectives of stakeholders on the challenges as well as the support for implementing survivorship care planning for this population. Rural Latina BC patients encounter complex challenges for survivorship care at cognitive, behavioral, and structural levels. Our findings demonstrates favorable opinions and support for the use of the SCP given its potential to serve as a comprehensive and portable tool that fills information gaps for patients, family, and HCPs, as well as enhances patients’ self-efficacy to promote proactive behavior in managing their cancer care.

## Figures and Tables

**Table 1:** Participant characteristics (N = 44).

Variables	M (SD)/N (%)		
	Patients (n = 12)	Family Caregivers (n = 8)	HCPs (n = 24)
**Age**	51.7 (9.5)	50.1 (19.9)	41.8 (13.7)
**Gender**			
Female	12 (100%)	6 (75%)	17 (70.8%)
Male		6 (25%)	7 (29.2%)
**Latina(o)/Hispanic**	12 (100%)	8 (100%)	16 (66.7%)
**Time of completion of cancer treatment (Month)**	15.6 (9.8)		
**Working full time (Yes)**			11 (91.7%)
**Years of Employment**			10.6 (10.0)
**Relationship to the patient**			
Adult children		3 (37.5%)	
Parent		2 (25%)	
Spouse		2 (25%)	
Sibling		1 (12.5%)	

**Table 2:** Additional quotes.

Themes	Additional Quotes
Theme 1 Lack of Knowledge of Treatment Information	“It can be something for them to see what type of medicine has been given or what type of test like MRI, PET scan or something like that, like the ones that they’ve already done” (FC 18). “I’ve seen patients who are getting chemotherapy for years. Then they come back one day and they say they didn’t know that they had cancer” (HCP 42). “The access of it [SCP] I think you’ll make them easier for them to go back and say oh, yeah this is what I had, or this is what happened, or this was a treatment plan that I received” (HCP 39).
Theme 2 Lack of Proactive Health Behavior	“When they are with the doctor, they just listen and listen and when they get out of the physician’s room they go like ‘So how many cycles do I need, what would be the next step’ … ” (HCP 2). “For example, an Anglo patient will come in with [what] they already Googled and researched. They already know the options they have so, it’s more of a dialogue back and forth with the physician versus the Latina sitting there and just go (stays quiet looking away)” (HCP 25). “I think that it (SCP) would help the patient know what questions to ask the next visit … ” (HCP 25).
Theme 3 Gaps in Information for Care Coordination	“We would have the kind of a situation medication accident in which doctors have to know that we are taking medicines. If we were to get into an accident (inaudible), we wouldn’t know or they might not know what types of treatment we have … ” (P 30). “ I realized that one doctor would not communicate with another doctor” (P 32). “They (patients) call all frustrated and saying, ‘I’m sick. Can you help? But you don’t know anything’. So, we feel bad for them and they feel bad for us, too. I have to tell them to come back in two weeks. Let me talk to your [doctor] … I don’t have enough information to tell you right now” (HCP 43).
Theme 4 Psychological Distress	“In my family there is no one with cancer. I did not know what to expect. I did not know where to go to ask … ” (P 11). “For some, it does worry them that they’re not seeing the doctor as often anymore because they’re scared that if the cancer comes back” (HCP 38). “Some of them they become happier after treatment and some of them are very dysphoric … they say, ‘Oh, I’m not going through again if this happened again’. So they’re very depressed and they’re very unstable mentally and physically. So, I try to refer them to group therapy … support that will help them because they have to hear from other cancer patients” (HCP 43).
Theme 5 Difficulty Retaining Health Information	“It (SCP) seems good to me … well, I would forget things, I forgot my treatments. For example, you ask me what treatment did you have? I don’t know” (P 14). “I think it will be great if a card (SCP) is implemented because we would have all the information in this booklet. Other than like the chemo, I think we no longer think. Well, then we tend to forget things, especially dates” (P 30). “We forget the date, what day they did this study, and well, everything is going to be there, with dates of treatment, what treatment was done … ” (FC 15). “Sometimes what I notice is that most of the patients are Spanish speaking and they have problems remembering the names of the doctors because they don’t know how to pronounce it. Another thing, they forget the names of the medications that it’s hard for them to remember. They just like saying ‘Oh, the pill that the doctor gave methe one that is for this … ‘ ” (HCP 2).

## Data Availability

The datasets generated during and analyzed during this study are available from the corresponding author on reasonable request.
